# A monogenean fish parasite, *Gyrodactylus chileani* n. sp., belonging to a novel marine species lineage found in the South-Eastern Pacific and the Mediterranean and North Seas

**DOI:** 10.1007/s11230-012-9379-2

**Published:** 2012-09-15

**Authors:** Marek S. Ziętara, Dar’ya Lebedeva, Gabriela Muñoz, Jaakko Lumme

**Affiliations:** 1 Department of Molecular Evolution, University of Gdańsk, Wita Stwosza 59, 80-308 Gdańsk, Poland; 2 Institute of Biology, Karelian Research Centre, RAS, Pushkinskaya 11, Petrozavodsk, Republic of Karelia, Russia; 3 Facultad de Ciencias del Mar y de Recursos Naturales, Universidad de Valparaíso, Casilla 5080, Reñaca, Viña del Mar, Chile; 4 Department of Biology, University of Oulu, P.O. Box 3000, 90014 Oulu, Finland

## Abstract

*Gyrodactylus* *chileani* n. sp. is the first *Gyrodactylus* species reported from Chile. It is an ectoparasite living on fins and skin of a small fish, the Chilean tidal pond dweller *Helcogrammoides chilensis* (Cancino) (Perciformes: Tripterygiidae). A phylogenetic analysis based on 5.8S+ITS2 of rDNA placed the new species close to marine *Gyrodactylus* species found in Europe: *G. orecchiae* Paladini, Cable, Fioravanti, Faria, Cave & Shinn, 2009 on gilthead seabream *Sparus aurata* L. from the Adriatic and Tyrrhenian Sea fish farms (Perciformes: Sparidae), and an undescribed species on the black goby *Gobius niger* L. from the North Sea (Perciformes: Gobiidae). A morphological description of the latter species is unavailable. These geographically distant parasite samples on different host families form a new well supported *Gyrodactylus orecchiae* lineage. Using molecular phylogenetics, it is shown that the marine species groups of *Gyrodactylus* may have a worldwide distribution.

## Introduction

The monogenean flatworm genus *Gyrodactylus* Nordmann, 1832 is one of the most species-rich genera. The number of described species was 409 in the latest checklist (Harris et al., 2004; see also GyroDB - http://www.gyrodb.net/), but the real number of species is estimated to be about 20,000 (Bakke et al., 2002), which is certainly an underestimate if the resolution of molecular methods is utilised and the classical criteria for delineating species are retained (Ziętara & Lumme, 2003).

Malmberg (1970) subdivided the genus into six subgenera. Two of these subgenera remain perfectly valid, i.e. those infecting Eurasian freshwater fish *G.* (*Limnonephrotus*) Malmberg, 1964 and *G.* (*Gyrodactylus*) Nordmann, 1832. The four other subgenera (*Metanephrotus* Malmberg, 1964*, Mesonephrotus* Malmberg, 1964, *Neonephrotus* Malmberg, 1964 and *Paranephrotus* Malmberg, 1964) are on the other hand paraphyletic or polyphyletic using molecular criteria (Matĕjusová et al., 2003; Vanhove et al., 2011).

The genus itself is not monophyletic and it is recognised to be a catch-all taxon within the Gyrodactylidae Beneden & Hesse, 1864 (Kritsky & Boeger, 2003). The genera: *Acanthoplacatus* Ernst, Jones & Whittington, 2001, *Diplogyrodactylus* Přikrylová, Matějusová, Musilová, Gelnar & Harris, 2009, *Fundulotrema* Kritsky & Thatcher, 1977, *Gyrdicotylus* Vercammen-Grandjean, 1960, *Gyrodactyloides* Bykhovskiy, 1947 and *Macrogyrodactylus* Malmberg, 1957, subgenera and species groups or single species, are all paraphyletic within the family Gyrodactylidae (see Vanhove et al., 2011).

The mechanism resulting in such a phylogeny is the basal radiation of *Gyrodactylus* (*sensu lato*) generating new species groups, some marine, some freshwater, and some inhabiting both environments. The latter are perhaps the most interesting, and include examples where the host lineage has moved from marine to freshwater, and the parasite has followed. The best known examples are two species, *G. lotae* Gusev, 1953 and *G. alexgusevi* Ziętara & Lumme, 2003, on the freshwater burbot *Lota lota* (L.), a gadid, and two species, *G. hrabei* Ergens, 1957 and *G. mariannae* Winger, Hansen, Bachmann & Bakke, 2008, on an inland cottid, *Cottus poecilopus* Heckel, in Europe. The nearest relatives of all four of these species are parasites of marine hosts (Ziętara & Lumme, 2003; Rokicka et al., 2009).

To enlarge the geographical range of the *Gyrodactylus* spp., we describe the first *Gyrodactylus* species from Chile, which was among 215 fish ectoparasitic species listed by Muñoz & Olmos (2007). Two other records of *Gyrodactylus* spp. on small intertidal fishes found in rocky areas, *Scartichthys* *viridis* (Valenciennes) (see Muñoz & Randhawa, 2011) and *Sicyases* *sanguineus* Müller & Troschel (see Muñoz & Zamora, 2011), await molecular analyses and further description. The species described below is also the first molecularly defined *Gyrodactylus* reported from the Southern Pacific. Together with two other species of *Gyrodactylus* from Europe, which are related by ITS rDNA, it forms a new marine species group, which has crossed the equator during its evolutionary history.

## Materials and methods

Fish were collected from rocky pools during low tides in July, 2009. Nineteen of 32 specimens were infected (prevalence 59.4%). Five ethanol-preserved worms were picked from the fins and skin of different hosts. The haptor was cut off, softened and cleared in 120 μg/ml proteinase K. It was then prepared for a microscopy on a slide with a saturated concentration of ammonium picrate in glycerine (Malmberg 1970). The remainder of the body was used for molecular analysis.

The molecular methods used in the present study were as previously described (Ziętara & Lumme, 2002, 2003; Ziętara et al., 2008). The ITS rDNA fragment encompassing ITS1-5.8S-ITS2 and short fragments of flanking 18S (15 bp) and 28S rRNA (9 bp) genes was amplified with primers *ITS1F* (5′-GTTTC CGTAG GTGAA CCT-3′) and *ITS2R* (5′-GGTAA TCACG CTTGA ATC-3′) and sequenced with two additional primers *ITS1R* (5′-ATTTG CGTTC GAGAG ACCG-3′) and *ITS2F* (TGGTG GATCA CTCGG CTCA-3′). The PCR program was 3 min at 95°C and 35 cycles of 40 sec at 94°C, 30 sec at 48°C and 1 min at 72°C, followed by a final elongation step of 7 min at 72°C and an indefinite hold at 4°C.

The phylogenetic placement of the new species was estimated by aligning the 5.8S+ITS2 segment of the ribosomal DNA with selected sequences published in GenBank. Only predominantly marine lineages of gyrodactylid species were included. The alignment was made using MUSCLE (Edgar, 2004) as implemented in MEGA5 (Tamura et al., 2011) and corrected manually. The phylogenetic tree was estimated from Maximum Composite Likelihood distances (Tamura et al., 2004) using the Neighbor Joining algorithm (Saitou & Nei, 1987) with the pairwise deletion option. The validity of the branches was evaluated by bootstrapping 500 replicates (Felsenstein, 1985). Bootstrap values lower than 70% were hidden.

For the species description, the preserved haptors were photographed and measured under a microscope using the measurements of Gusev et al. (1985). Measurements of *G. orecchiae* Paladini, Cable, Fioravanti, Faria, Cave & Shinn, 2009 by Paladini et al. (2009) were included for comparison. The North Sea species on *Gobius niger* remains undescribed (Huyse et al., 2003).

## Phylogenetic characterisation using the 5.8S+ITS2 rDNA fragment

**Fig. 1:**
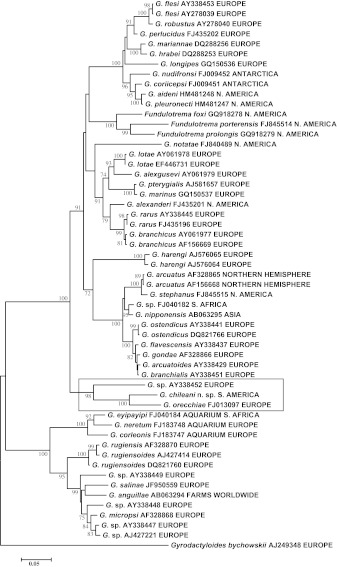
Hypothetical phylogenetic position of *Gyrodactylus chileani* n. sp. based on 5.8S+ITS2 rDNA sequences of marine (or of marine origin) gyrodactylids. Bootstrap support less than 70% is omitted. *Gyrodactylus* sp. FJ040182 from *Liza* *richardsonii* (Smith); *Gyrodactylus* sp. AY338452 from *G*obius *niger* (see Huyse et al., 2003); *Gyrodactylus* sp. AY338449 from *Pomatoschistus* *norvegicus* (Collett), a species provisionally called *G*. cf. *longicactylus* (see Huyse et al., 2003); *Gyrodactylus* sp. AJ427221 from *Pomatoschistus* *lozanoi* (de Buen), a species provisionally called *G*. cf. *micropsi* (see Huyse & Volckaert, 2002); and *Gyrodactylus* sp. AY338447 from *P*. *lozanoi*, a species provisionally called *G*. cf. *micropsi* (see Huyse et al. 2003)

As this report focuses on the new species from Chile and its nearest relatives, we present a phylogeny based on 5.8S+ITS2 sequences of marine representatives of *Gyrodactylus* (Fig. 1), omitting all exclusively freshwater subgenera and species groups, which have already been well analysed by Vanhove et al. (2011). The new species from Chile (JQ045347) clustered with high (98%) bootstrap support with two geographically distant species. These were *G. orecchiae* (FJ013097), described from Mediterranean cultured gilthead seabream *Sparus aurata* (Perciformes: Sparidae) by Paladini et al. (2009), and an undescribed species sequenced and discussed by Huyse et al. (2003) from the black goby *Gobius niger* (Perciformes: Gobiidae, AY338452) in the North Sea. The relatedness of these two European species was noted by Paladini et al. (2009), but they did not place the species into a wider phylogenetic context. The phylogeographic coverage of this lineage is now extended significantly to include the Southern Hemisphere and the Pacific Ocean.

The complete ITS rDNA (including ITS1+5.8S rDNA+ITS2) sequence is not available for *Gyrodactylus* sp. from *Gobius* *niger* (see Huyse et al., 2003) and therefore the complete ITS segment of the rDNA was aligned only for *Gyrodactylus* *orecchiae* and the new species from Chile. The Maximum Composite Likelihood distance was estimated to be 15.4% between these two species, but it must be stressed that the ITS1+5.8S+ITS2 segments of the rDNA are not optimal for distance estimation, due to problems with the reliable alignment of ITS rDNA, from less related *Gyrodactylus* species with distances above 10%.

## Remarks on the systematic relationships of the new taxon

*Subgeneric position.* On the basis of the molecular sequence of the ITS region of the ribosomal DNA, the new species cannot be placed in any of the subgenera previously suggested by Malmberg (1970), which are already rendered poly- and paraphyletic by the inclusion of molecular data (Fig. 1). The Chilean *Gyrodactylus* species is placed within the mixed basal group of “short ITS” (Cable et al., 1999) species. The “short ITS” clade receives 100% bootstrap support in Fig. 1 as the sister group of the *G. rugiensis, G. micropsi* and *G. eyipayipi* species groups, which are the only marine groups (100% bootstrap support) among the “long ITS clade”, and a sister clade of the subgenus *G*. (*Limnonephrotus*) (not demonstrated in Fig. 1, but see the phylogeny of Vanhove et al., 2011).

*New species group.* The new species clusters with *G. orecchiae* from the Mediterranean Sea (100% bootstrap support) and with an undescribed species on black goby from the North Sea. The three species cluster together with 98% bootstrap support and are relatively basal in the badly resolved clade (91% bootstrap support) of mostly marine, but also some derived freshwater, species. These species have previously been assigned to *G.* (*Mesonephrotus*), *G.* (*Metanephrotus*) and *G.* (*Paranephrotus*), or are not assigned at all. The considerable geographical spread of the three species assigned to this new *G. orecchiae* species lineage and the systematic diversity of their hosts suggest that a more comprehensive sampling may add numerous new species to this group.

## *Gyrodactylus chileani* n. sp.

*Type-host*: *Helcogrammoides chilensis* (Cancino) (Perciformes: Tripterygiidae); local name ‘Trombollito de tres aletas’ (three-finned trombollito).

*Type-locality*: Tidal ponds at Las Cruces, Valparaiso (33°30′S, 71°37′W), El Tabo, Chile. Sampling date July 22nd, 2009.

*Site*: Fins and skin.

*Type-material*: Slides of 5 isolated opisthaptors and 1 complete specimen of *G. chileani* n. sp. were deposited in Finnish National History Museum in Helsinki University. Holotype: MZH 118095; paratypes: MZH 118096 (complete specimen); MZH 118097-MZH 118100 (isolated haptors).

*Molecular data*: Definitive identification is based on the nucleotide sequence of the internal transcribed spacers of the nuclear ribosomal DNA. The accession number for the ITS1-5.8S-ITS2 and short flanking segments of 18S and 28S rDNA from the holotype specimen is JQ045347. The holotype was cut in two, the haptor was mounted on a slide, and the remainder was used for DNA analysis. The ITS rDNA sequence was repeated from all of the five specimens that were measured.

*Etymology:* The name of the species is derived from the country name, as it is the first *Gyrodactylus* species described from Chile.

### Description (Figs. 2, 3; Table 1)

**Fig. 2:**
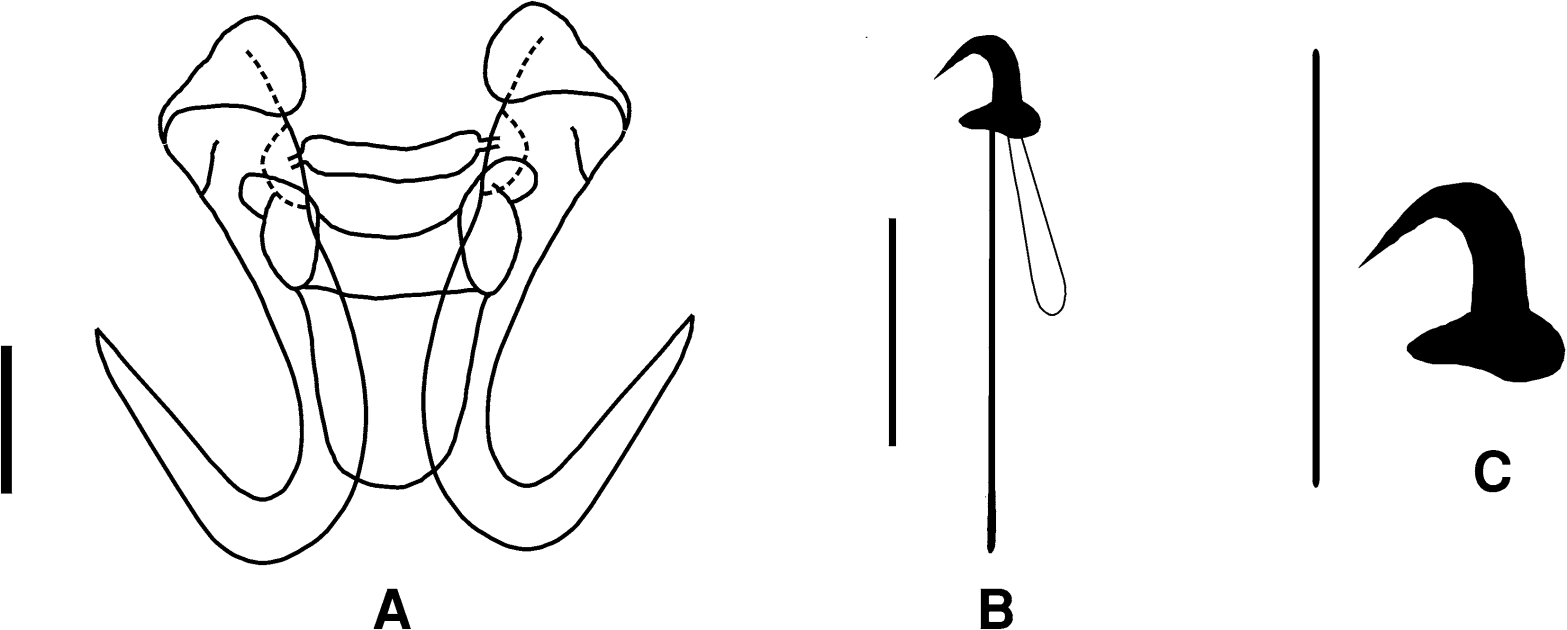
Haptoral morphology of *Gyrodactylus chileani* n. sp. A. hamuli, dorsal and ventral bars; B. marginal hook; C. marginal hook sickle. *Scale-bar*: 10 μm

**Fig. 3:**
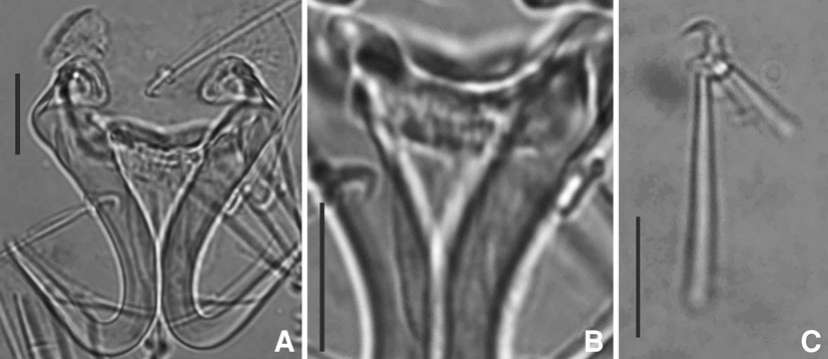
Photographs of *Gyrodactylus chileani* n. sp. Holotype. A. hamuli; B. ventral bar; C. marginal hook. *Scale-bar*: 10 μm

[Five specimens were measured. Table 1 contains ranges and mean values of all measurements. Total length of anchor not measured as root is folded.] Shaft 23–27 μm; point 17–20 μm. Ventral bar length 16–20 μm, width 3–5 μm; membrane length 9–12 μm. Dorsal bar length 12–17 μm, width 2–3 μm. Marginal hook total length 21–23 μm; sickle length 4–5 μm. Shapes of hamuli resemble those of *G. orecchiae* and also those of *G. jirovecii* species goup of Eurasian freshwaters, which belong, according to DNA evidence, to subgenus *G*. (*Limnonephrotus*). Hamulus roots folded and points extend almost to half length of shafts (Figs. 2A, 3A). Ventral bar with much smaller processes than in *G. orecchiae* (Figs. 2A, 3B). Dorsal bar with narrow attachments (Fig. 2A). Marginal hooks clearly more delicate than in *G. orecchiae;* toe triangular in shape, not rhomboid as in *G. orecchiae*. Shaft of marginal hook points downwards and extends beyond toe. Heel pronounced, but smaller than in *G. orecchiae* (Figs. 2B, C, 3C).

**Table 1:** Measurements (in micrometres) of the haptoral hard parts of *Gyrodactylus chileani* n. sp.

Specimens	Hamuli		Dorsal bar		Ventral bar			Marginal hook	
	Shaft length	Point length	Length	Width	Length	Width	Membrane length	Total length	Sickle length
All (5)	23–27 (25)	17–20 (18)	12–17 (14.5)	2–3 (2.5)	16–20 (18)	3–5 (4)	9–12 (11)	21–23 (22)	4–5 (4.4)
Holotype	27	19	14	2	16	5	12	22	4.5
*G. orecchiae*	21	16	16	2	22	5	11	18	3.5

Mean values in parentheses. Measurements of *G. orecchiae* Paladini, Cable, Fioravanti, Faria, Cave & Shinn, 2009 from Paladini et al. (2009)

## Discussion

An overt phylogenetic revision of monogenean parasites of fishes was published by Perkins et al. (2009), with an informative title “Looks can deceive…”. This seems to be a general rule among this class of parasites, certainly extending to the specific level among *Gyrodactylus* spp. With respect to the geographical coverage, the few molecular phylogenetic studies which have been attempted during the last decade are encouraging but far from satisfactory in terms of the strategic planning of a systematic revision (Cable et al., 1999, 2005; Harris & Cable, 2000; Ziętara et al., 2000, 2002, 2008; Huyse & Volckaert, 2002, 2005; Ziętara & Lumme, 2002, 2003, 2004; Boeger et al., 2003; Huyse et al., 2003, 2004, 2006; Matĕjusová et al., 2003; Huyse & Malmberg, 2004; LeBlanc et al., 2006; Malmberg et al., 2007; Kuusela et al., 2008; Rokicka et al., 2009; Vanhove et al., 2011). Due to the small fraction of the genus analysed in the above works, little can be said about its systematics. Additionally, a global systematic revision is certainly premature and unwarranted when less than 2% of the suspected species have been found and described; nevertheless, as the genus appears not to be monophyletic (Kritsky & Boeger, 2003, Vanhove et al., 2011), some kind of revision is needed. An alternative strategy towards the global systematics of the family Gyrodactylidae could be an extraction of well-supported monophyletic lineages based on their DNA, such as the nine lineages visible in present phylogeny, and their further supplementation by morphological characters, but leaving the genus *Gyrodactylus* as polyphyletic until all of its evolutionary lineages are resolved.

Such a new lineage is revealed in the present study. It consists of only three *Gyrodactylus* species at present. The hosts of the species belong to the order Perciformes, but the suborders and families differ. These are: *Gobius niger* (black goby) [suborder Gobioidei: family Gobiidae]; *Helcogrammoides chilensis* [Blennioidei: Tripterygiidae]; and *Sparus aurata* (gilthead seabream) [Percoidei: Sparidae]. The parasites on *S. aurata* were recorded in fish farms from Croatia and Albania (Paladini et al., 2009) or Italy (Paladini et al., 2011b), so it is not known whether the fish is the natural host of *Gyrodactylus orecchiae.* Considering the species richness of these host families (e.g. Hickey et al., 2009), we may expect that the three parasite species mentioned here are just a small subsample. They are all marine monogeneans and the geographical distance indicates that it is likely that at least the coastal waters of all seas contain related parasite species.

The phylogenetic hypothesis constructed in the present study demonstrates that the marine *Gyrodactylus* species groups, and in some cases even the species, are quite widely distributed. The *G. orecchiae* lineage extends from the Mediterranean and North Sea to the South-Eastern Pacific. *Gyrodactylus arcuatus* Bychowsky, 1933 has been confirmed on three-spined sticklebacks *Gasterosteus aculeatus* L. from Puget Sound (USA), Iceland, the Mediterranean and Black Seas, the Sea of Japan, Belgium (Cable et al., 1999; Ziętara et al., 2000), Finland (Ziętara et al., 2002, 2008), Norway (Ziętara et al., 2008; Hansen et al., 2012), Poland (Ziętara et al., 2000), Russian Karelia (Ziętara et al., 2008), Sweden (Ziętara et al., 2000; Huyse et al., 2003) and the UK (Cable et al., 1999; Ziętara et al., 2000). Its very close relatives include *Gyrodactylus nipponensis* Ogawa & Egusa, 1978 from Japan (Hayward et al., 2001) and *G. stephanus* Mueller, 1937 from the NW Atlantic (King & Cone, 2009) on other host fishes. On three-spined sticklebacks, *G. alexanderi* Mizelle & Kritsky, 1967 has also been confirmed using molecular data in both Atlantic (Hansen et al., 2012) and Pacific populations (Rokicka et al., 2009), but *G*. *avalonia* Hanek & Threlfall, 1969 and *G*. *canadensis* Hanek & Threlfall, 1969 await molecular confirmation.

The *G. eyipayipi*, *G. micropsi* and *G. rugiensis* species groups also have a very interesting connection across the equator: *G. eyipayipi* Vaughan, Christison, Hansen & Shinn, 2010 has been recorded on greater pipefish *Sygnathus acus* L. in South African waters (Vaughan et al., 2010), and *G. micropsi* Gläser, 1974 and *G. rugiensis* Gläser, 1974 on the gills and fins of *Pomatoschistus* *microps* Krøyer, respectively, have been reported from European waters (Ziętara et al., 2002; Huyse et al., 2003, 2006). The related *G. corleonis* Paladini, Cable, Fioravanti, Fiara & Shinn, 2010 and *G. neretum* Paladini, Cable, Fioravanti, Fiara & Shinn, 2010 (Paladini et al. 2010) were found on pipefish *Syngnathus* *typhle* L. and *S*. *scovelli* Evermann & Kendall, respectively, from aquaria in Europe. *Gyrodactylus salinae* Paladini, Huyse &Shinn, 2011 on *Aphasius* *fasciatus* Valenciennes has a Mediterranean distribution (Paladini et al., 2011a), and several species on gobies, provisionally referred to as *G*. cf. *micropsi* and *G*. cf. *longidactylus,* occur in the North Sea (Huyse & Volckaert, 2002; Huyse et al., 2003). Lastly, *G. anguillae* Ergens, 1960 is a cosmopolitan species on eels (see Hayward et al. 2001).

It has been already reported that the two Antarctic gyrodactylids, *G. coriicepsi* Rokicka, Lumme & Ziętara, 2009 and *G. nudifronsi* Rokicka, Lumme & Ziętara, 2009, are related to the European *Gyrodactylus* fauna, indicating the evolutionary continuum of the marine species in the Northern and Southern Hemispheres (Rokicka et al., 2009). This lineage also accommodates two recently described species, i.e. the European *G*. *longipes* Paladini, Hansen, Fioravanti & Shinn, 2011, reported as a mixed infection with *G*. *orecchiae* from *Sparus* *aurata* in the waters of Bosnia-Herzegovina and Italy (Paladini et al., 2011b), and the North American *G. aideni* Mullen, Cone, Easy & Burt, 2010 along with *G. pleuronecti* Cone, 1981, a species recently tagged by ITS rDNA (Mullen et al., 2010).

So far, the few *Gyrodactylus* species sampled from the coasts of the Southern Pacific and Southern Atlantic have been found to have quite close relatives in the species groups studied in the Northern Hemisphere. Connections between the Northern Pacific and Northern Atlantic are even tighter, including common species such as *G. arcuatus*. This finding supports the hypothesis of Boeger et al. (2003) suggesting the origin of viviparous gyrodactylids in South American waters and a further expansion from that continent via the marine environment. Such a scenario requires a mixing of the marine gyrodactylid fauna as demonstrated in the present paper.
